# Infantile Congenital Mesoblastic Nephroma Leading to Multi-Systemic End-Organ Disease

**DOI:** 10.7759/cureus.30513

**Published:** 2022-10-20

**Authors:** Liana Grosinger, Irim Salik, Bhupen Mehta

**Affiliations:** 1 Department of Anesthesiology, Westchester Medical Center, Valhalla, USA

**Keywords:** malignant hypertension, pediatric anesthesiology, pediatric solid renal tumors, infantile tumor, abdominal compartment syndrome (acs), congenital mesoblastic nephroma

## Abstract

Congenital mesoblastic nephroma (CMN) is a rare infantile abdominal tumor that is highly curable with early surgical intervention. However, chronic, unrecognized tumor burden can cause significant compression of local vascular and solid structures, resulting in multi-systemic end-organ dysfunction. In this case report, we describe the effects of chronic abdominal compartment syndrome in an infant due to a solid renal tumor and its anesthetic implications.

## Introduction

Although congenital mesoblastic nephroma (CMN) is a rare pediatric renal tumor, it is the most common renal neoplasm in neonates less than one month in age [1]. The differential diagnosis for pediatric solid renal tumors includes Wilms, CMN, clear cell sarcoma, ossifying renal tumor of infancy, and malignant rhabdoid tumor. CMN has been classified into three subtypes: cellular, classic, and mixed; these can be differentiated histologically from each other. Management of CMN with a radical nephrectomy is often curative, particularly for the classic subtype [2]. Although disease presentation, classification, and management of CMN have been well described, there is little information on its anesthetic implications. In this case report, we discuss the multi-systemic complications and anesthetic considerations in an infant with CMN.

## Case presentation

The patient was a three-month-old male, born full-term after an uncomplicated pregnancy, who presented with persistent emesis and decreased oral intake in the setting of increasing abdominal distention. Abdominal distension had been noted at one month of age, but no medical intervention had been sought. In the emergency department, his vital signs were as follows - heart rate: 132 beats per minute, BP: 200/100 mmHg, respiratory rate: 33 breaths per minute, SpO_2_: 97% on room air, temperature: 36.6 °C, and weight: 5.1 kg. Physical exam was notable for a distended abdomen, palpable non-mobile abdominal mass in the left lower quadrant extending past midline to the right abdomen, marked edema in the bilateral upper and lower extremities, and a full anterior fontanelle with papilledema. Labs were notable for blood urea nitrogen (BUN) of 40 mg/dL, creatinine of 1.4 mg/dL, troponin of 0.77 ng/mL, and B-type natriuretic peptide (BNP) of 3342 pg/mL.

**Figure 1:**
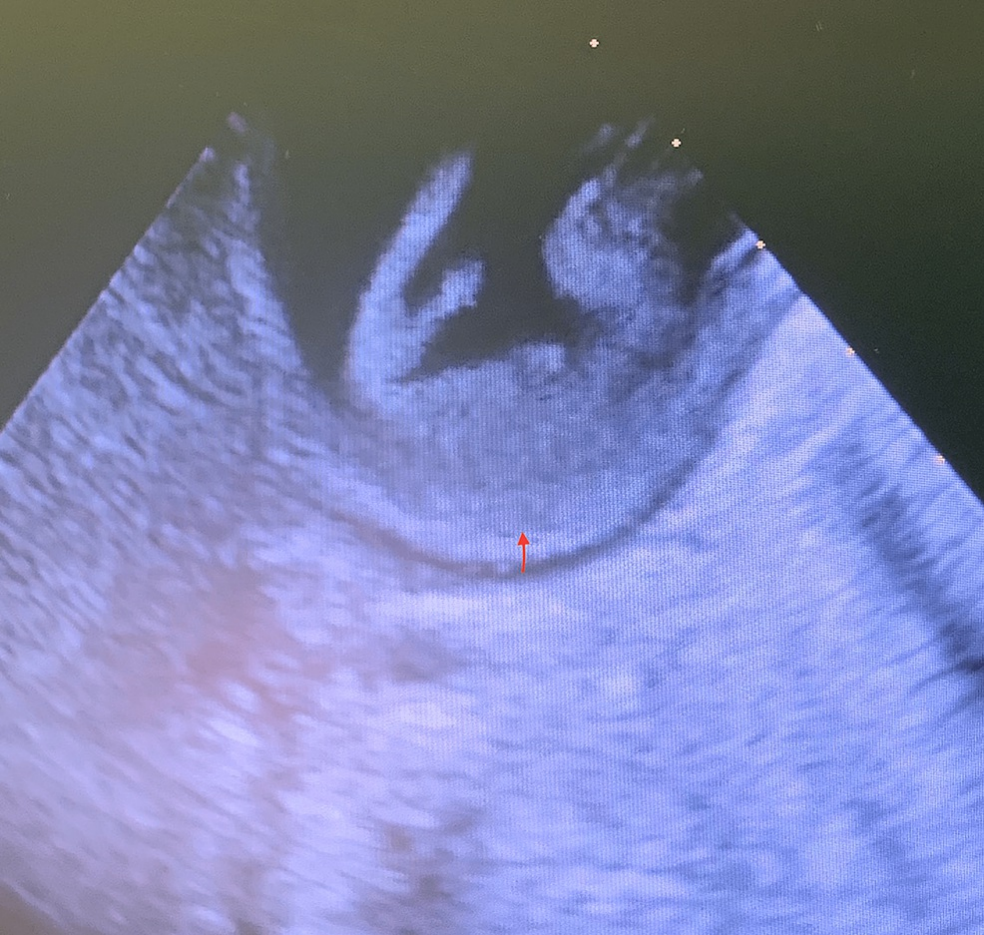
Cardiac POCUS parasternal short-axis view showing severe left ventricular hypertrophy (arrow) POCUS: point-of-care ultrasound

**Figure 2:**
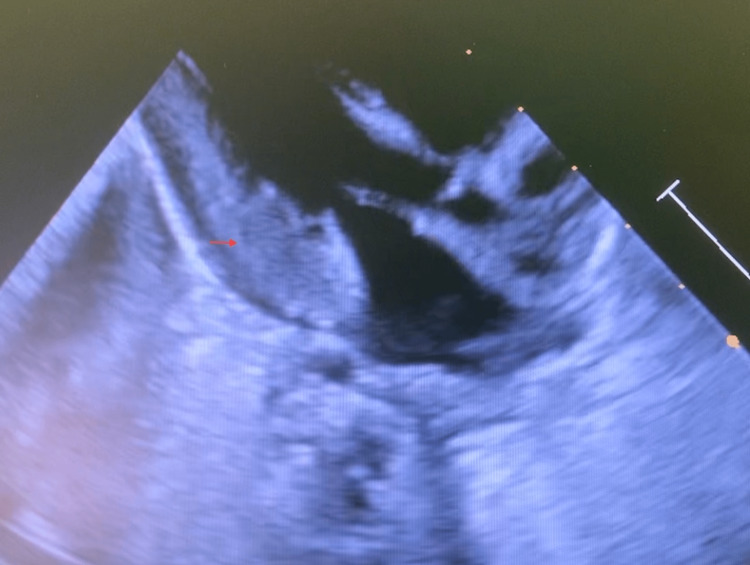
Cardiac POCUS parasternal long-axis view showing severe left ventricular hypertrophy (arrow) POCUS: point-of-care ultrasound

Given the patient’s severely elevated blood pressure, the need for invasive line placement, and the imaging required to further characterize the mass, the decision was made to admit the patient to the pediatric intensive care unit (PICU) and intubate. Prior to induction, cardiac point-of-care ultrasound (POCUS) showed severe concentric left ventricular hypertrophy with depressed left ventricular function and apical dyskinesis (Figures 1, 2). A pre-induction right radial arterial line was placed for close hemodynamic monitoring. The patient was adequately pre-oxygenated, and a rapid sequence induction was done with 3 µ/kg fentanyl and 1.2 mg/kg rocuronium. The patient was intubated without difficulty with a Miller 1 blade; a grade 1 Cormack-Lehane view was obtained and a 3.0 cuffed endotracheal tube was placed. No gastric contents were visualized in the oropharynx. Following intubation, blood pressure continued to be severely elevated at 200-250/100-150 mmHg. Esmolol and nicardipine infusions were started, with marked improvement.

**Figure 3:**
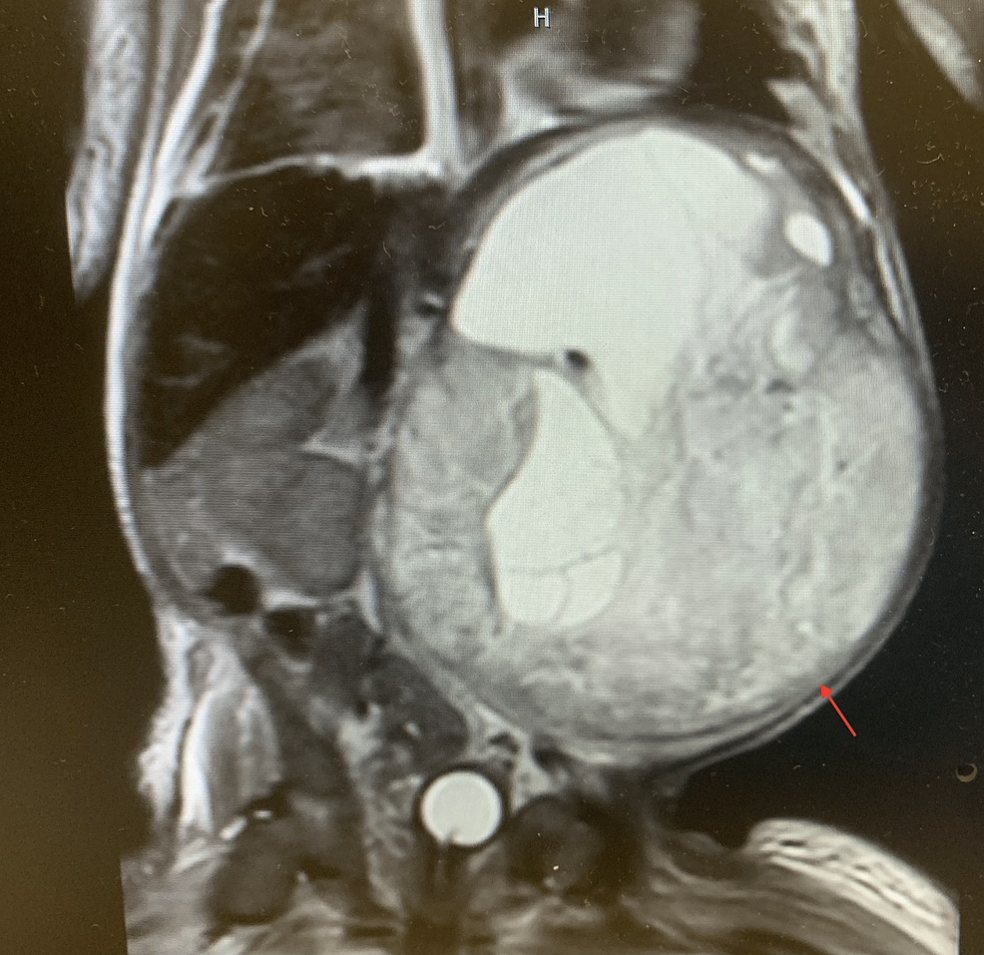
Abdominal MRI coronal image showing tumor burden causing rightward aortic displacement (arrow) MRI: magnetic resonance imaging

**Figure 4:**
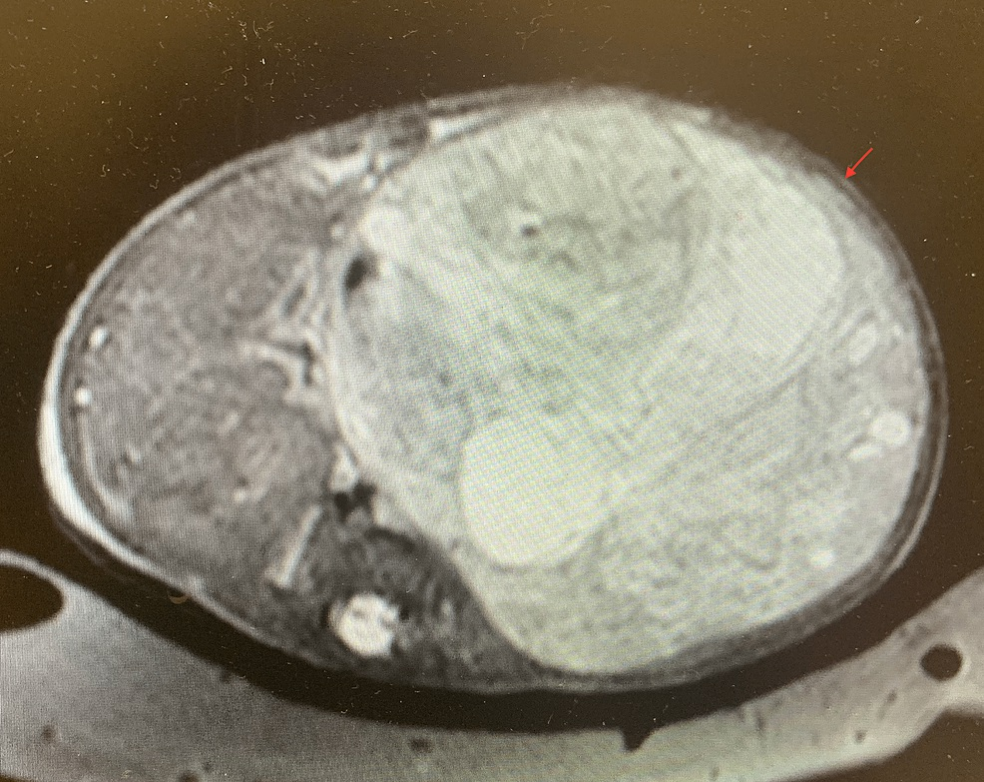
Abdominal MRI axial image displaying the large tumor (arrow) MRI: magnetic resonance imaging

CT abdomen showed a very large heterogeneous renal mass with extension to the left abdomen, measuring 12.4 x 12.2 x 10.9 cm with associated mass effects on the liver, gallbladder, right kidney, and bowel loops. Abdominal MRI revealed rightward aortic displacement and tumor invasion of the left renal vein (Figures 3, 4). A head CT revealed hydrocephalus with dilated lateral and third ventricles. Oliguric acute kidney injury (AKI) persisted despite trials of Lasix and mannitol. Hence, a double-lumen 7 Fr non-tunneled central venous catheter was placed and continuous veno-venous hemodialysis (CVVHD) was started for acute renal failure complicated by volume overload. Blood pressure continued to improve after starting CVVHD.

The surgical service planned the resection of the renal mass following appropriate diagnostic imaging. The right radial arterial line remained in place and the central venous catheter used for CVVHD was used intraoperatively for central access. Preoperative vital signs were as follows - HR: 125, BP: 105/65, SpO_2_: 98% on pressure control ventilation (RR: 18, PEEP: 5, FiO_2_: 40%), and temperature: 37 °C. The patient was assigned an American Society of Anesthesiologists (ASA) physical status classification of 4. Anesthesia was maintained with sevoflurane and fentanyl and non-depolarizing neuromuscular blocking drugs were re-dosed appropriately. An external ventricular drain (EVD) was placed for hydrocephalus and increased intracranial pressure (ICP) prior to the renal mass resection. Norepinepherine was initiated at 0.05 mcg/kg/min following renal mass resection to maintain afterload in the setting of a BP level of 62/36 and HR of 194. An open left radical nephrectomy and a right renal biopsy were performed without complication. The estimated blood loss was 50 ml, and the total urine output was 5 ml over the course of four hours. During surgery, the patient was transfused 100 ml packed red blood cells and 50 ml platelets; he was given 10 ml albumin and a total of 85 ml plasmalyte. The patient remained intubated and was transported to the PICU.

In the immediate postoperative period, there was a significant improvement in the patient’s hemodynamics, and the vasopressor was weaned off. The patient’s cardiac markers, including BNP and troponin, trended down. The patient was extubated on postoperative day (POD) 15, delayed secondary to multiple failed extubation attempts due to limited respiratory reserve. CVVHD was continued postoperatively and discontinued on POD 6. An EVD was left in place for three weeks for cerebrospinal fluid (CSF) drainage; subsequently, an intraparenchymal hematoma developed with intraventricular extension, at which time a ventriculoperitoneal shunt was placed. The patient was eventually discharged to a rehabilitation facility on POD 62 with close outpatient follow-up planned with the neurosurgery and nephrology teams.

## Discussion

Our case report describes an infant with benign CMN with resultant chronic abdominal compartment syndrome, leading to malignant hypertension, acute renal failure, and intracranial hypertension requiring permanent CSF diversion. The patient presented with elevated troponin and BNP levels, with severe left ventricular hypertrophy in the setting of untreated hypertension, likely of multi-factorial etiology. This may have been reflective of the exceptionally high afterload from the renal mass and compression of the surrounding great vessels, along with elevated renin levels and renal artery stenosis. Coronary blood flow may have been compromised secondary to significant ventricular hypertrophy, causing apical dyskinesis. Cerebral ventriculomegaly and hydrocephalus were likely caused by increased intra-abdominal pressure and venous obstruction from the tumor, leading to elevated ICP.

Given the extensive multi-system effects of this large tumor, intubating and providing intraoperative care for this patient posed great challenges for the anesthesiologist. During induction, in an attempt to avoid pulmonary aspiration, a rapid-sequence induction was planned due to the patient’s large abdominal mass and persistent emesis. Prior to imaging, elevated ICP was suspected given the physical exam findings of a full anterior fontanelle and papilledema. Given the patient’s elevated ICP, the hemodynamic goals of induction consisted of maintaining systemic blood pressure in order to maintain adequate cerebral perfusion pressure and avoiding hypoxia. Prior to induction, cardiac POCUS showed severe concentric left ventricular hypertrophy with depressed left ventricular function and apical dyskinesis. Maintaining systemic vascular resistance was critical in ensuring adequate coronary blood flow to an already compromised myocardium. Establishing adequate intravenous access and invasive monitoring was essential for the intraoperative course. The radial arterial line remained in place because of the potential for significant hemodynamic instability during tumor handling due to mechanical compression of surrounding structures, particularly the great vessels. Central access was needed for the use of vasopressors in order to maintain systemic vascular resistance. Blood products were readily available in case of large intraoperative blood loss. Delayed diagnosis of CMN can lead to significant morbidity and mortality in infants, requiring close communication between the surgical and anesthetic teams during tumor resection.

Similar cases of abdominal compartment syndrome and its anesthetic implications have been described in the literature, but mostly with regard to the multi-systemic effects of a Wilms tumor. In a review article by Whyte and Mark Ansermino, the authors engage in a thorough discussion of the anesthetic considerations with a focus on intraoperative management [3]. The importance of being aware of the possible hemodynamic instability from mechanical obstruction of venous return is highlighted. While this article does not refer to CMN, it describes a similar scenario with a different large pediatric abdominal mass.

CMN is considered a highly curable neonatal malignancy that requires early surgical intervention and management of potential recurrence. It is a rare tumor with low malignant potential, representing 3% of all pediatric renal tumors. Complete surgical resection with radical nephrectomy in the first week of life commonly leads to a disease cure. Neonates exhibit improved survival rates in comparison to fetuses, and there are few chronic complications associated with this tumor [4]. Chemotherapy should be reserved for recurrent tumors that are not amenable to resection. Most infants present with local disease, and nephrectomy is considered the standard of care [5]. Chemotherapeutic regimens have not been strictly prescribed due to the rarity of the tumor, but treatment with alkylating drugs, anthracyclines, and a Wilms-like approach using vincristine and dactinomycin have shown efficacy.

Gooskens et al. found that the median age at diagnosis of CMN was less than one month in 63% of patients, and prenatal detection was reported in 16% of patients [6]. There was a slight male predilection at a ratio of 1.5:1. Infants most commonly presented with an abdominal mass (76% of cases), followed by hypertension (19%) and hematuria (11%). Most tumors are detected antenatally based on a palpable abdominal mass. Although the neonates are generally stable at presentation, up to 71% of cases can be associated with perinatal complications including hydrops fetalis, preterm delivery, nonreassuring fetal heart rate patterns, and intrauterine fetal death [7]. Other symptoms can include but are not limited to polyhydramnios, elevated renin levels, and hypercalcemia. In the series described, congenital anomalies were reported in 11 patients, genitourinary malformation in six patients, gastrointestinal complications in two patients, and one case each of polydactyly, hydrocephalus, and Beckwith-Wiedemann syndrome [6]. Within the sample, mortality was approximately 4%, and either associated with underlying disease or chemotherapy treatment, including postoperative sepsis or neutropenia, postoperative necrotizing enterocolitis, and excessive intraoperative blood loss [6].

Disease relapse was documented in 4% of patients; as either local recurrence or metastasis to the lung, liver, and bone within 12 months of primary tumor resection. Patients with metastasis to the bone, brain, peritoneum, and cardiac structures had a 100% mortality rate. Factors that worsen patient outcomes include stage III disease and a cellular subtype. Furtwaengler et al. found that higher relapse rates were associated with infants who presented at greater than three months of age, likely due to a more aggressive cellular subtype in older infants [8]. Infants should be followed clinically and radiologically for 18 months after tumor resection to evaluate for recurrence. Whittle et al. have reported a rare instance of marked tumor attenuation and complete resection without recurrence following eight months without treatment [9]. Mata et al. have described an infant with CMN who developed microcephaly, cerebral atrophy, hypotonia, and tetraparesis within the first year of life following a right nephroureterectomy on the 10th day of life [10]. Mata’s case report highlights how hemodynamic perturbations due to tumor burden can lead to devastating neurologic compromise due to prolonged hypoxia.

## Conclusions

Based on our findings, infants with CMN should be promptly diagnosed and an appropriate index of suspicion should be maintained in infants with a significant history and physical findings. As evidenced by this case report, even benign CMN can result in multi-organ dysfunction. The anesthesiologist must be prepared to manage the perioperative complications associated with chronic abdominal compartment syndrome that can occur in patients with a large tumor burden. Safe anesthetic care is predicated on the identification and management of systemic complications in this vulnerable patient population.
